# Calculation of Vibrational Circular Dichroism Spectra Employing Nuclear Velocity Perturbation or Magnetic Field Perturbation Theory Using an Atomic-Orbital-Based Linear Response Approach

**DOI:** 10.1021/acs.jpca.5c01344

**Published:** 2025-05-01

**Authors:** Ravi Kumar, Sandra Luber

**Affiliations:** Department of Chemistry, University of Zürich, Winterthurerstrasse 190, 8057 Zürich, Switzerland

## Abstract

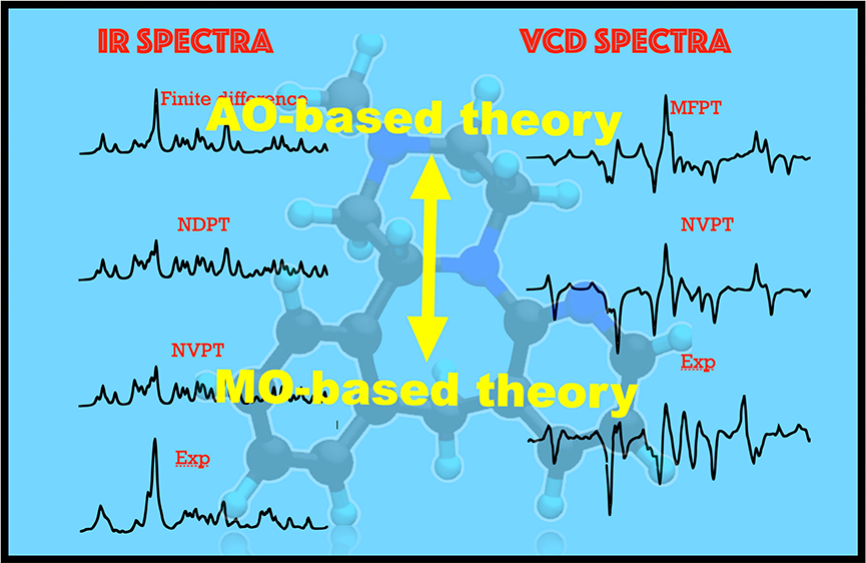

We present the implementation of an AO-based solver for vibrational circular dichroism (VCD) spectra calculations, employing nuclear velocity perturbation theory using velocity-gauge included in atomic orbitals or magnetic field perturbation theory using gauge-including atomic orbitals. The implementations are done within the Gaussian and plane waves framework in the CP2K package. The previously implemented approaches in CP2K are based on MO-solvers, which solve the Sternheimer equation for linear response. Our AO-solver implementations were validated against the MO-solver by performing VCD calculations for the R-enantiomer of mirtazapine. Additionally, we extended the AO-based solver implementation to nuclear displacement perturbation theory in order to calculate infrared absorption spectra. The AO-based solver produced spectra that matched exactly with the MO-based results, confirming the accuracy of the implementation. This allows for efficient calculations of vibrational properties, further extending the capabilities of CP2K for large molecular systems.

## 1 Introduction

Circular dichroism (CD) is a spectroscopic technique that measures the differential absorption of left and right circularly polarized light by optically active chiral molecules. Vibrational Circular Dichroism (VCD) is an extension of CD into the infrared (IR) and near-IR regions of the electromagnetic spectrum, providing insight into vibrational transitions. VCD spectra, often referred to as IR spectra for chiral molecules, are particularly useful in characterizing the stereochemistry and molecular structure of these systems.^1,2^ While the IR spectra of chiral molecules do not differentiate between enantiomers, VCD spectra exhibit equal intensity but opposite signs for their mirror images. This characteristic makes VCD a powerful tool for distinguishing between enantiomers^3^ and has important applications in drug industries.^4^ VCD spectroscopy has been widely utilized as a characterizing tool to probe the structure of materials and molecules.^5,6^ Other applications are in understanding the supramolecular filament chirality^7^ and applications in the solid state (see e.g. refs (8) and (9)). Additionally, being highly sensitive to intermolecular interactions,^10^ this makes it useful for studying various phenomena, such as reactant-catalyst binding and the transfer of chirality during catalytic processes.^11,12^

The observable parameter for experimentally measured VCD intensities is the rotational strength, which can be calculated in terms of electric dipole moment (μ) and magnetic dipole moment (*m*) derivatives.^1,13,14^ In CP2K, analytic derivatives of these moments with respect to nuclear positions and nuclear velocities, respectively, have been implemented following density functional perturbation theory (DFPT) approaches.^15,16^ The analytic derivative of μ with respect to nuclear positions, also called atomic polar tensors (APTs), have been implemented for both periodic and nonperiodic molecular systems using nuclear displacement perturbation theory (NDPT) done previously in the group.^17^ Additionally, atomic axial tensors (AATs), which refer to magnetic moment derivatives, were implemented using nuclear velocity perturbation theory (NVPT) with velocity atomic orbitals (VAOs),^15,18^ and magnetic field perturbation theory (MFPT) with gauge-independent atomic orbitals (GIAOs).^15^ All these perturbation theories in CP2K are implemented within the DFPT framework using the Gaussian and plane-wave approach (GPW) with pseudopotentials.^19^ In addition, nonlocal pseudopotentials were included in both MFPT^20^ and NVPT^21^ approaches.

The current implementation of VCD spectra calculations in CP2K is based on a molecular orbital (MO) response solver, where perturbed MO coefficients are calculated by solving the Sternheimer equation using linear perturbation theory, followed by the computation of properties. However, using an MO-based approach presents some challenges, especially when dealing with large molecular systems. For such systems, the diagonalization of the Kohn–Sham (KS) or Hartree–Fock (HF) matrix scales cubically and requires significant memory resources,^22^ limiting the applicability of these methods. An alternative to MO-based theories is to work entirely in the atomic orbital (AO) basis, avoiding explicit use of MO coefficients. Various linear-scaling algorithms have been proposed.^22−27^ This work adopts the optimization of the one-electron density matrix and energy through the exponential parametrization technique developed by T. Helgaker et al.^22^ This technique is favored for its computational efficiency, as sparse matrix operations lead to near-linear scaling of computational costs. These operations can be efficiently performed using the Distributed Block Compressed Sparse Row (DBCSR) matrix library in CP2K,^28^ which enables linearly scalable computations on large systems.

For perturbed properties’ calculations, HF energy derivatives were derived by Pulay,^29,30^ and later implemented by Gaw and Handy,^31^ as well as Thomsen.^32^ The present work, however, employs the exponential parametrization of the AO density matrix derived by Larsen et al.,^33,34^ which has been shown to be an effective approach for optimizing the density matrix. A similar approach, developed by Ochsenfeld and colleagues,^35,36^ utilizes McWeeny’s purification scheme for the optimization of the AO density matrix. In CP2K, the AO-based response solver has been implemented for Harris functional energy correction, and the details of MO and AO solvers’ scaling and efficiency have been discussed comprehensively.^37^ In a recent study by Ravi and Luber,^38^ this solver was used to compute electric dipole polarizabilities for both periodic and nonperiodic systems.

In the present work, we extend this solver for other perturbations, namely Nuclear Displacement Perturbation Theory (NDPT), which deals with real and symmetric perturbations, as well as Nuclear Velocity Perturbation Theory (NVPT) and Magnetic Field Perturbation Theory (MFPT), both of which involve imaginary and antihermitian perturbations. Unlike in the case of electric fields and the Harris functional, in these perturbations the perturbed overlap integral, S^(1)^, is nonzero, which necessitated modifications to the previously implemented AO-based solver to include the S^(1)^ term, and the derivation of APTs and AATs undergoes essential modifications. These modifications are crucial to ensure the expressions of properties are accurately reformulated in terms of response density matrices.

The rest of the paper is organized into eight sections. Section 2 presents a general theoretical framework for VCD spectra calculations using both NVPT and MFPT. A brief derivation of the response density matrix in AO-based response theory is provided in Section 3. Section 4 discusses NVPT in the context of VCD calculations using both molecular orbital (MO) and atomic orbital (AO)-based approaches, while Section 5 covers MFPT with both solvers. IR absorption spectra calculation using both response solvers employing NDPT and NVPT is discussed in Section 6. Computational details are outlined in Section 7, followed by the results in Section 8, with conclusions in Section 9. Unless stated otherwise, all results and equations presented in this paper are presented in atomic units (*ℏ* = *m*_*e*_ = *e* = 1/4πϵ_0_ = 1). For magnetic properties, we follow the CGS unit convention.

## 2 Theory of VCD Spectra Calculation

In double harmonic approximation, a VCD spectra is determined by calculating the rotational strength, which depends on calculation of APTs and AATs. For the *i*-th normal mode, the rotational strength (RS), *R*_*i*_, of a vibrational transition is defined as^1,13,15^
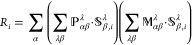
1where  and  are the APT and AAT elements, respectively, for λ atom and α and β are the Cartesian directions. In eq 1,  represents the mass-weighted transformation matrix from Cartesian to normal mode coordinates, which in CP2K is computed by seminumerically. In this paper, we employ both NVPT and MFPT to calculate the RS and the theory presented is for Kohn–Sham (KS) DFT. First, we will discuss RS by means of the NVPT.

The APTs are defined as the derivative of the expectation value of the electric dipole moments with respect to the nuclear positions (in the length representation). In the NVPT approach, we adopt its velocity representation^15^ and derivatives are taken with respect to the nuclear velocity. Thus, the electronic and nuclear contribution to APTs in velocity representation are given by
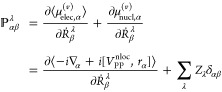
2where the derivative is taken with respect to the nuclear velocities *Ṙ*_β_^λ^. The first term in eq 2 consists of the velocity form of the electric dipole operator, which can be derived from the relation of Hyper-virial theorem and Heisenberg time derivative.^21,39^ −*i*∇ is the linear momentum operator and *V*_PP_^nloc^ is the nonlocal pseudopotential, that is very common to use in electronic structure calculations to reduce the computational cost.^19^ In the second term, *Z*_λ_ is the charge (or valence charge when pseudopotentials are used) of λ atom.

The AAT in NVPT (, in eq 1) is defined as the derivative of the expectation value of the magnetic dipole moment operator with respect to the nuclear velocities. The electronic and nuclear contribution to AAT are defined as
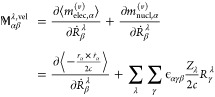
3where we write  as electronic contribution to the magnetic dipole moment operator in the velocity representation. ***r*** and ***ṙ*** represent the position and velocity operators, respectively, and *c* is the velocity of light in vacuum. In the second term of eq 3, ϵ_αγβ_ is the Levi–Civita symbol and sum goes over Cartesian directions γ and atoms λ.

In the MFPT formalism, AATs expression is defined as follows: Theoretically, the calculation of VCD spectra from NVPT is related to the one from MFPT,^40^ where the electronic part of AATs is defined as the second derivative of the KS energy with respect to the nuclear velocity and magnetic field,
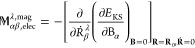
4
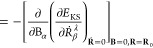
5where both formulas for AATs in eqs 4 and 5 are equivalent and in case of KS DFT, the partial derivatives of the KS energy with respect to nuclear velocity **Ṙ** and magnetic field **B** can be interchanged. **R**_0_ denote the equilibrium geometry’s coordinates. Eq 5 can be considered as the length form of the electronic part of AATs. In complete adiabatic approximation, the total Hamiltonian is defined as^40^
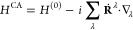
6where the second term is the perturbation operator added to the unperturbed KS-Hamiltonian *H*^(0)^ which can be derived from the kinetic energy operator of the λ nuclei.^15^ In eq 6, **Ṙ**^λ^ and ∇_λ_ are the nuclear velocities of λ–th atom and gradient operator acting on electronic wave function, respectively. Moreover, the second term in eq 6 is a first-order contribution beyond the Born–Oppenheimer approximation (see ref (41) for details). Inserting eq 6 in eq 5, one can derive the expression of AAT in MFPT as^40^
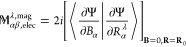
7where Ψ is the total ground state electronic wave function.

## 3 Theoretical Details of Atomic-Orbital-Based Linear Response Theory

In KS-DFT formalism, the unperturbed KS-Hamiltonian operator consists of the following terms
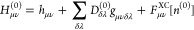
8where μ, ν are the indices of the spin–orbital basis and *n*^(0)^(***r***) is the electron density as a function of real-space coordinate ***r***. *h*_μν_ contains kinetic energy and nuclear potential energy terms and is the one-electron part of the Hamiltonian. **D**^(0)^ is the unperturbed one-electron density matrix defined as *D*_μν_^(0)^ = ∑_*j*_*f*_*j*_*C*_μ*j*_^(0)^*C*_*j*ν_^(0)^, assuming real MO coefficients, where *C*_μ*j*_^(0)^ is the MO coefficient element of the *j*th MO orbital, and *f*_*j*_ is the occupation number of the *j*th MO. The second term in eq 8 is the electrostatic potential energy term^34^ and *F*_μν_^XC^[*n*^(0)^] is the exchange-correlation (XC) energy functional. The greek indices {μ, ν, δ, λ ...} represent the AOs and roman indices {*i*, *j*, *k*, ...} represent the occupied MOs. Please note that we skipped the ^(0)^ superscript for quantities in eq 8 except of *H*, *D* and *n*, indicating an unperturbed quantity. Due to nonorthogonal basis function χ_μ_(**r**), the overlap integral term **S**^(0)^ is introduced whose element is written as *S*_μν_^(0)^ = ⟨χ_μ_|χ_ν_⟩. Furthermore, the electron density can be defined as *n*^(0)^(**r**) = ∑_μν_*D*_μν_^(0)^*S*_μν_^(0)^, for the given overlap matrix **S**^(0)^.
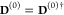
9a
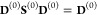
9b
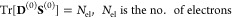
9c
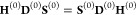
9d

The density matrix calculated from the SCF calculation is a valid density matrix and satisfies all the conditions given in eqs 9a–9d. However, in the presence of external perturbation, the density matrix no longer remains valid and does not satisfy these conditions. Hence, we employ the exponential parametrization scheme introduced by T. Helgaker et al.^22^ to ensure its validity. According to this parametrization, a density matrix can be formed from **D**^(0)^ using the following expression:^34^
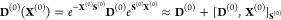
10where **X**^(0)^ is an anti-Hermitian matrix which can be separated into a real antisymmetric part and an imaginary symmetric part depending on the real and imaginary perturbation. The term [**D**^(0)^, **X**^(0)^]_**S**^(0)^_ represents **D**^(0)^**S**^(0)^**X**^(0)^ – **X**^(0)^**S**^(0)^**D**^(0)^. The exponential term in eq 10 has been expanded up to the second term using the Baker-Campbell-Hausdorff (BCH) expansion.^42^

Furthermore, there may be some redundant density matrices that do not change, i.e., **D**^(0)^(**X**^(0)^) = **D**^(0)^(**0**) = **D**^(0)^, and these must be eliminated to avoid unnecessary divergence or slow convergence issues.^42^ These issues can be avoided by applying the projection operator :^22^
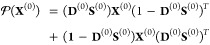
11where, **D**^(0)^**S**^(0)^ and (**1** – **D**^(0)^**S**^(0)^) are the projection operators operated on the occupied and virtual orbital space, respectively.

To calculate the second order perturbed properties (such as APTs and AATs in the present case), first order response density matrix is required to be evaluated. The response equation for determining the perturbed density matrix can be derived as follow: Differentiating eq 10 with respect to the perturbation parameter *x*_*a*_ at **X** = **0**(34) gives
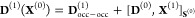
12where the superscript ^(1)^ represents first-order perturbed quantities. Throughout the paper, a perturbed quantity *g*^(1)^ with respect to parameter *x*_*a*_ is defined by *g*^(1)^ = d*g*(*x*_*a*_)/d*x*_*a*_. **D**_occ–occ_^(1)^ in eq 12 is calculated by projecting the derivative of the idempotency condition (9b) onto the occupied-occupied space as^34^
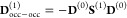
13Other contributions, such as the unoccupied-unoccupied blocks are zero and the occupied-unoccupied blocks are undetermined. Next, the response equation is derived from differentiating the variational condition given in eq 9d with respect to **x**
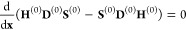
14Expanding all the contributing terms in eq 14 and simplifying further, we obtain the final working equation to calculate **X**^(1)^(34,38)
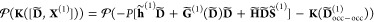
15where the quantities with (∼) are given in the orthogonal Löwdin basis, transformed according to the following relation:^43^
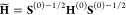
16
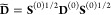
17The quantities are transformed into the Löwdin basis to avoid inconsistencies between covariant and contravariant tensors.^37,43^ In eq 15, the derivative of the KS Hamiltonian operator (from eq 8) is written as
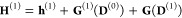
18where **h**^(1)^ is the perturbed one-electron Hamiltonian, **G**^(1)^(**D**^(0)^) contains the perturbed two-electron terms with the unperturbed density matrix, and **G**(**D**^(1)^) represents the two-electron terms involving the perturbed density matrix. To simplify the response equation further, we defined **K**(**A**) = *P*[**G**(**A**)**D**^(0)^**S**^(0)^ + **H**^(0)^**AS**^(0)^] and to combine the negative terms, we introduced another term *P*[**A**] = **A** – **A**^†^, where **A** is a general quantity.^34^

The response eq 15 is solved using the preconditioned conjugate gradient method with appropriate preconditioner. In this work, the preconditioner employed is derived from an additional conjugate gradient approach detailed by S. Coriani et al. in ref (33), and implemented in CP2K.^37^

## 4 Rotational Strength Using NVPT

In this section, we discuss the derivation of the RS calculation applicable for nonperiodic systems using both MO-based and AO-based response theories.

### 4.1 Using MO-Based Response Theory

The expression to compute APTs and AATs needed for the RS calculation requires perturbed MO-coefficients and density matrices in MO-based and AO-based response theories, respectively. In NVPT, in order to calculate the properties independent of the choice of gauge, and to include the velocity gauge factor, the velocity atomic orbitals (VAO), originally described by Nafie,^40^ are employed,
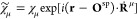
19where χ_μ_ is the atom-centered AO basis function. For simplicity, we omitted the dependence of the parameters such as electron positions **r**, nuclear positions **R** and **Ṙ** in the equation. VAOs are spatial origin dependent from origin **O**^sp^. Here, we define some useful functions that will be needed while solving the response equation. The derivative of χ̃_μ_ with respect to nuclear velocity (at **Ṙ**^μ^ = 0) can be derived as^15^
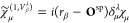
20where δ_μ_^λ^ denotes that the basis function, χ_μ_, is centered at the λ-th atom and superscript ^(1,*V*_β_^λ^)^ represents the derivative with respect to the nuclear velocity of λ-th atom in the β Cartesian direction. Using the relation from eq 20, the perturbed overlap matrix derivative (*S*_μν_^(1,*V*_β_^λ^)^) can be obtained
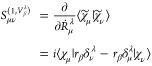
21

A response density matrix *D*_μν_^(1,*V*_β_^λ^)^ or perturbed MO coefficient matrix *C*_μ*j*_^(1,*V*_β_^λ^)^ needs to be calculated to obtain APTs. In MO-based theories, a Sternheimer equation is solved to calculate *C*_μ*j*_^(1,*V*_β_^λ^)^ and using them *D*_μν_^(1,*V*_β_^λ^)^ can be determined^15^
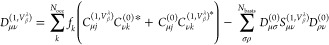
22*k* is summed over all the occupied MOs and Greek indices ρ, σ go over all AO basis functions. {*C*_ν*k*_^(0)^} and {*C*_μ*j*_^(1,*V*_β_^λ^)^} are the unperturbed and perturbed MO coefficients, respectively. In CP2K within GPW and KS-DFT, the unperturbed single particle Hamiltonian operator is given by
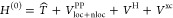
23where we skipped the ^(0)^ superscript for quantities on the right side. *H*^(0)^ consists of the single electron kinetic energy operator *T̂*, pseudopotential *V*_loc+nloc_^PP^ having local and nonlocal components, and *V*^H^ and *V*^xc^ are Hartree and the exchange–correlation potential operators, respectively. The KS-orbital |ψ_*j*_⟩, eigen function of *H*^(0)^, is expanded in terms of basis functions |χ_μ_⟩ as |ψ_*j*_⟩ = ∑_μ_*C*_*j*μ_^(0)^|χ_μ_⟩. MO-coefficients {*C*_*j*μ_^(0)^} are obtained by solving the Roothan-Hall equation
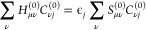
24Perturbing all the quantities in eq 24 with respect to the external perturbation and keeping the linear terms in perturbative parameter, we can derive the response equation to determine the *C*_*j*ν_^(1,*V*_β_^λ^)^ as follow^15,17^
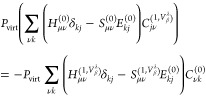
25where *P*_virt_ represents the projection operator acting on the unoccupied orbitals, defined as *P*_virt_ = 1 – ∑ _*j*_^N_occ_^|ψ_*j*_⟩⟨ψ_*j*_|. {*C*_*j*ν_^(1, *V*_β_^λ^)}^ are the perturbed MO coefficients represented in NVPT. In order to construct the Sternheimer equation, it is needed to have, among others, expressions for **H**^(1)^**C**^(0)^ and **S**^(1)^, where **H**^(1)^ and **S**^(1)^ represent the perturbation Hamiltonian and perturbed overlap matrix, respectively. Taking the derivative of eq 6 with respect to *Ṙ*_β_^λ^ and multiplying it with **C**^(0)^ we get^15^
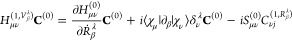
26where the first term is the derivative of unperturbed KS-Hamiltonian matrix with respect to nuclear velocity. The second and third terms are consequences of the derivatives of the perturbation Hamiltonian (second term in eq 6). The expression for **S**^(1,*V*_β_^λ^)^ is already given in eq 21.

From eq 26, it can be noted that the last term contains *C*_ν*j*_^(1,*R*_β_^λ^)^, which is the response MO coefficient element with respect to nuclear position. *C*_ν*j*_^(1,*R*_β_^λ^)^ is calculated using nuclear displacement perturbation theory (NDPT), which has been implemented in CP2K.^17^ Detailed derivation can be found in refs (17) and (44).

By substituting eq 26 into eq 25 and solving it using the preconditioned conjugate gradient scheme, we obtain *C*_*j*ν_^(1,*V*_β_^λ^)^. However, solving eq 25 for *C*_*j*ν_^(1,*V*_β_^λ^)^ accounts only for the response corresponding to the contribution from the occupied-unoccupied block of the response density matrix. The response corresponding to the unoccupied-unoccupied block remains zero.^45^ The response of the occupied MOs (*C*_μ*j*,occ_^(1,*V*_β_^λ^)^) that corresponds to the occupied-occupied block of the density matrix response is incorporated without explicitly solving the response equation, as it is accounted for through the perturbed overlap integral term **S**^(1,*V*_β_^λ^)^, as given in^15^
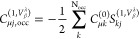
27In the following, we denote the total perturbed MO coefficient element as *C*_μ*j*_^(1,*V*_β_^λ^)^. As a result, the electronic part of APTs in NVPT can be determined from eq 2:^17^
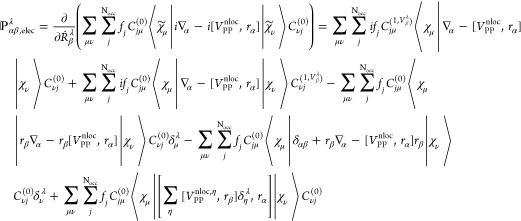
28and electronic contribution to AATs is evaluated using eq 3 as
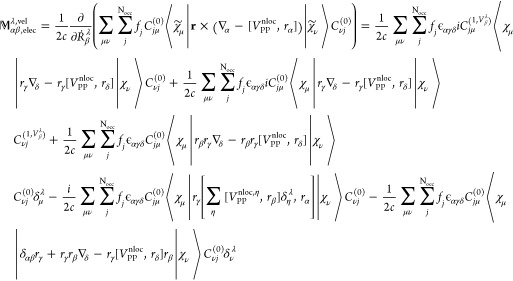
29

Eq 29 is similar to APT expression in eq 28 with the difference that there is an additional multiplication by position operator *r*_γ_ in eq 29. Additionally, in the APT expressions in eq 28, we have omitted spatial origin dependence of position operator because position operator in VAO is gauge dependent and we write (**r**_β_ – **O**_β_^sp^) →**r**_β_ for clarity. Similarly, we have omitted the magnetic gauge origin dependence in eq 29, as the magnetic dipole operator is also gauge-dependent, and replaced (**r**_γ_ – **O**_γ_^mag^) →**r**_γ_. For a detailed discussion regarding gauge origin dependence, we refer to ref (15).

### 4.2 Using AO-Based Response Theory

All the necessary quantities and parameters in AO-basis required for AO-based response theory have been derived and explained in the previous section. From the AO-based linear response eq 15, the perturbation Hamiltonian matrix **H**^(1,*V*_β_^λ^)^ and the perturbed overlap matrix **S**^(1,*V*_β_^λ^)^ are provided in eqs 26 and 21, respectively. In order to build the **H**^(1,*V*_β_^λ^)^, it is necessary to compute the response density matrix elements *D*_μν_^(1,*R*_β_^λ^)^ using the AO-solver with NDPT, along with the unperturbed density matrix elements, *D*_μν_^(0)^ = ∑_*j*_*f*_*j*_*C*_μ*j*_^(0)^*C*_*j*ν_^(0)^, assuming real MO coefficients. The details of the Hamiltonian matrix derivative, *H*_μν_^(1,*R*_β_^λ^)^, and the overlap matrix derivative, *S*_μν_^(1,*R*_β_^λ^)^ which are required to construct the response equation, are provided in ref (17).

Since the perturbation Hamiltonian in NVPT is imaginary, the perturbed response density matrix *D*_μν_^(1,*V*_β_^λ^)^ is also imaginary and anti-Hermitian. Consequently, the trial matrix or initial guess for calculating *D*_μν_^(1,*V*_β_^λ^)^ is anti-Hermitian as well. The response eq 15 is solved to evaluate **X**^(1)^ using the conjugate gradient method by performing uncoupled response calculation. To accelerate the calculation and prevent divergence, a *multilevel* preconditioner^37^ has also been implemented in CP2K, which is based on an additional conjugate gradient approach as described in ref (33). As can be seen in eq 15, we obtain **X**^(1)^ by solving this response equation. However, to compute the complete response density matrix, given in eqs 12 and 13, it is essential to include the contribution from the occupied-occupied block **D**_occ–occ_^(1)^, which is obtained by calculating **S**^(1,*V*_β_^λ^)^ using eq 21.

After obtaining *D*_μν_^(1,*V*_β_^λ^)^, we can calculate the APTs using NVPT in AO based formalism. For that we did not explicitly derive the AATs expression; instead, we expressed eq 28 in terms of density matrices, which takes the form:^15^
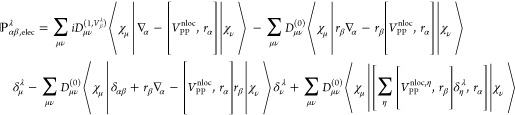
30and electronic contribution to AATs is
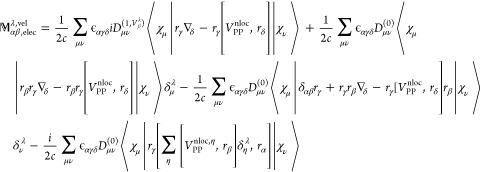
31

## 5 Rotational Strength Using MFPT

In this section, we discuss the calculation of RS using both MO-based and AO-based response theories employing MFPT. We can use length representation of APT expression, required to determine the RS from NDPT,^17^ to make the RS expression independent of the gauge shift.^15^

### 5.1 Using MO-Based Response Theory

As mentioned in section 2, an alternative method for calculating AATs is by using MFPT. The total Hamiltonian in the presence of external magnetic field is
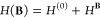
32where *H*^**B**^ is the perturbation Hamiltonian which is defined as^15,46^
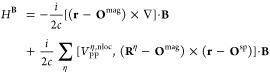
33where **O**^mag^ and **O**^sp^ are magnetic and spatial origin, respectively. In the second term, the summation is taken over pseudoatoms. With using the approximated wave function or finite basis sets, the results depend on the choice of gauge origin.^36^ To overcome this problem, gauge-independent atomic orbitals (GIAOs) have been suggested which are discussed in refs (15) and (46).

Now we can derive perturbed overlap integral and Hamiltonian matrix in order to build the Sternheimer eq 25 using the GIAOs. The overlap derivative matrix with respect to magnetic field at **B** = 0 can be obtained by
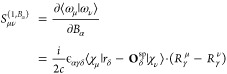
34where magnetic field is applied in the α Cartesian directions. **O**^mag^ term cancels and eq 34 is free from magnetic gauge origin. Similarly, the matrix elements of the gauge transformed Hamiltonian derivative can be evaluated^15^
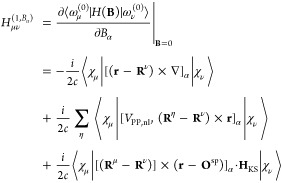
35Using the formulation with gauge transformation is advantageous because the matrix elements calculated are independent of **O**^mag^ but still have dependency on **O**^sp^. Additionally, as evident in the second line of eq 35, the matrix element depends only on the difference of coordinates instead of the absolute coordinates. After calculating *H*_μν_^(1,*B*_α_)^ and *S*_μν_^(1,*B*_α_)^ in eqs 34 and 35, respectively, we can evaluate *C*_*j*μ_^(1,*B*_α_)^ by solving eq 25 using preconditioned CG method. In MFPT, the imaginary perturbation does not affect electron density, leading to uncoupled response equations. This simplifies the computational process allowing for efficient solution of the equations.

We can thus calculate the electronic contribution to the AATs following eq 5(15)
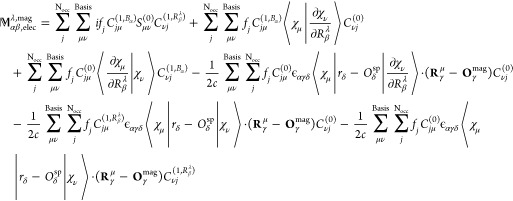
36where the summation goes to all atoms γ and Cartesian coordinates δ. In the AAT expression (36), **O**^mag^ and **O**^sp^ appear explicitly, hence AAT result has dependency on the choice of **O**^sp^ as can be seen from eqs 34 and 35.

### 5.2 Using AO-Based Response Theory

In MFPT, AATs are calculated using the AO-based solver in a similar manner to the NVPT case. To construct the AO response eq 15, **H**^(1)^ and **S**^(1)^ are taken from eqs 35 and 34, respectively. Furthermore, the eq 15 is solved to calculate **X**^(1)^ using preconditioned CG method and uncoupled response equations are solved. The complete response density matrix *D*_μν_^(1,*B*_α_)^ is achieved by adding the contribution from eq 13, as shown in eq 12.

Analogous to eq 36, the electronic contribution to the AATs is written in density matrix form^15^
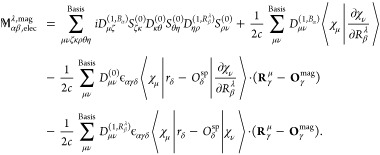
37In eq 37, all terms except the first one on the right-hand side were straightforward to write in density matrix form from eq 36. However, the first term in eq 36 does not explicitly involve the unperturbed MO coefficient matrix. Therefore, we take help of the normalization identity of the MO wave function, **C**^(0)*T*^**S**^0^**C**^(0)^ = **1**, and modify the first term as follows:
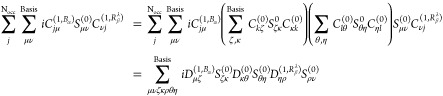
38where indices *k*, *l*, *j* denote the MO coefficients and Greek symbols ζ, κ, η and θ represent the basis functions. Eq 38 is equivalent to first term in eq 36 in MO-basis.

## 6 IR Spectra from NDPT and NVPT Using AO- and MO-Based Response Solvers

IR absorption spectra can be computed using various theoretical approaches, such as finite differences, NDPT and NVPT. In the finite differences method, IR intensities are obtained by calculating the electric dipole moment for slightly displaced nuclear positions in both positive and negative Cartesian directions (or alternatively with respect to normal coordinates). This approach approximates the response of the molecular system by evaluating electric dipole differences due to these incremental displacements. In CP2K, a numerical derivative using finite differences is available in conjunction with vibrational analysis calculations. Recently, analytical methods employing NDPT and NVPT were also implemented in CP2K in refs (17) and (15), respectively. NDPT directly evaluates the derivative of electric dipole moments in length form with respect to nuclear displacements via a perturbative approach, where a symmetric perturbation Hamiltonian generates coupled response equations for each nuclear coordinate. NVPT, which uses an imaginary perturbation Hamiltonian corresponding to nuclear velocities, calculates IR intensities by evaluating electric dipole derivatives in velocity form in an uncoupled manner. Both NDPT and NVPT use response theory.

An important parameter for IR spectra calculations is the atomic polar tensor , as discussed in previous sections. The observable quantity, dipole strength (DS_i_), using DFPT can be obtained by
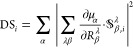
39where the term  is an element of the APT with *R*_β_^λ^ being an element of the nuclear position and  has same meaning as given in Section 2. μ_α_ represents the electric dipole moment operator defined by
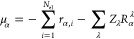
40where the terms on right-hand side represent the electronic and nuclear part of the electric dipole operator, respectively. Here, we have used the length representation of the electric dipole operator, which is suitable for nonperiodic systems. In NDPT, by following an MO-based response solver and using the total response MO coefficients *C*_ν*j*_^(1,*R*_β_^λ^)^ calculated in Section 4, we can evaluate the electronic contribution to the APT using the expression
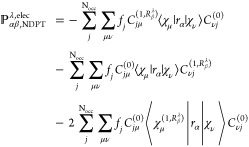
41where χ_μ_^(1,*R*_β_^λ^)^ is the perturbed basis function.^17^

Using AO-based response solver, the APT expression in terms of response density matrix with electric dipole operator defined in eq 40 can thus be calculated as
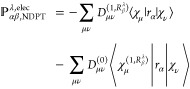
42where *D*_μν_^(1,*R*_β_^λ^)^ is an element of the perturbed density matrix in NDPT. In NVPT, APTs can be calculated using the velocity dipole operator defined in eq 2, following the expression given in eq 28 for origin independent results. Further, the equivalent expression for calculating APTs using the AO-based solver is given in eq 30.

Within MFPT, we employ the length representation of the electric dipole operator, whereas in NVPT, we use its velocity representation. Despite being different forms of the operator, both yield identical results, but only in the complete basis set limit.^15,16,38,47,48^

## 7 Computational Details

**Figure 1:**
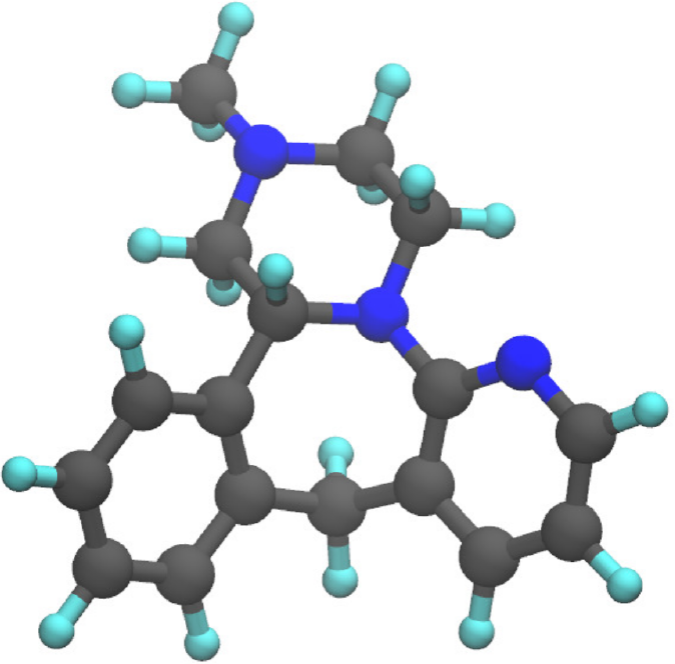
Optimized structure of R-enantiomer of mirtazapine molecule. Black, cyan, and blue color represent carbon, hydrogen, and nitrogen atoms, respectively.

We use as an example system the R-enantiomer of mirtazapine (C_17_H_19_N_3_) (see Figure 1). The geometry of the structure was optimized using TZV2P-GTH basis set^49^ and Goedecker–Teter–Hutter (GTH)-type pseudopotentials^50^ using the CP2K program within the Quickstep environment^19^ at the Kohn–Sham-DFT level. Maximum force and root-mean-square force convergence criteria were set to 3 × 10^–5^ a.u. and 1.5 × 10^–5^ a.u., respectively, and the PBE^51^ exchange correlation functional was employed. The energy convergence criterion was set to 1.08 × 10^–6^ a.u., and the charge density cutoff was set to 600 Ry. The optimized molecular structure is shown in Figure 1. Afterward, vibrational analysis calculation was carried out in the harmonic approximation using three point difference formula with increments using the default value 1 × 10^–2^ a.u. in each Cartesian direction of all the atoms. SCF convergence and charge density cutoff were set to 3.08× 10^–7^ a.u. and 600 Ry, respectively with same basis set and functional pseudopotential as used for geometry optimization. Thereafter, the calculated Hessian matrix was diagonalized to obtain vibrational frequencies. Furthermore, electric dipole moment derivative with respect to nuclear coordinates was computed numerically to obtain IR intensities from finite differences. We also computed APTs analytically using DFPT, following ref (17), and IR intensities from both approaches as well as from the AO-solver were compared.

Next, we performed APT and AAT calculations to simulate the VCD spectrum using NDPT,^17^ NVPT, and MFPT^15,16^ implementations with DFPT in CP2K, employing convergence threshold for the response solver of 5 × 10^–12^ a.u. and the basis set mentioned above. We chose the velocity gauge origin, magnetic gauge origin, and reference origin to be (0, 0, 0), following the common origin gauge approach for our calculations. All DFPT calculations were also performed using AO-based solver with analogous inputs as used for the MO-based solver calculations. We used a Lorentzian broadening based on the following expression:
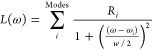
43where *L*(ω) is the spectral line and broadening was applied to RS *R*_*i*_. ω_*i*_ is the angular frequency corresponding to normal mode *i* and *w* stands for the full width at half-maxima which was set to 12 cm^–1^ for all calculated spectra. Summation has been taken over all the modes obtained from vibrational analysis.

**Figure 2:**
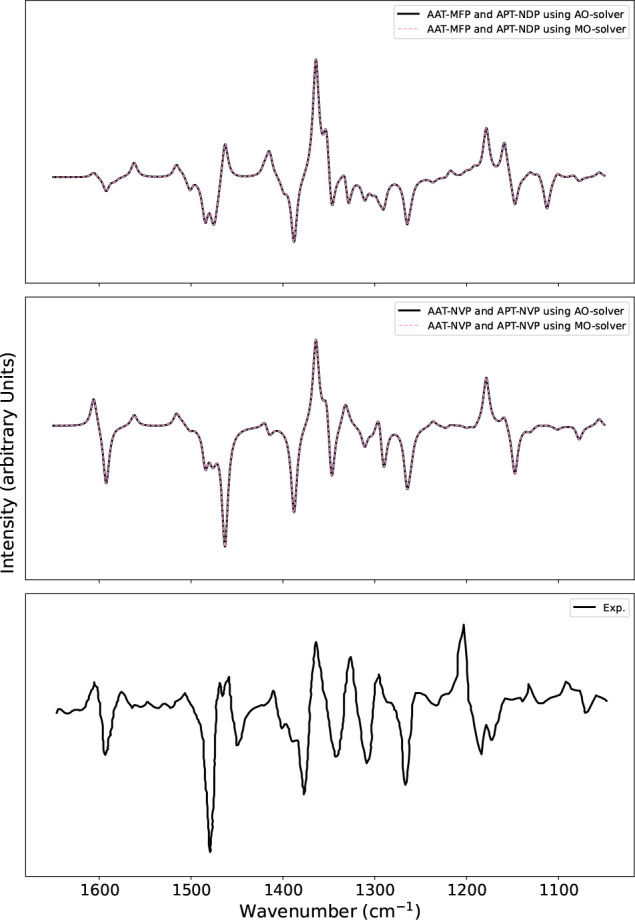
Plots illustrating the VCD spectra calculated using NVPT and MFPT with MO-based and AO-based response solvers, respectively. The spectra are compared to the experimental spectrum that was digitized from ref (52) (bottom plot). Middle plot: AAT-NVP and APT-NVP represent spectra where both AAT and APT were computed using NVPT. Top plot: AAT-MFP and APT-NDP represent spectra where AATs were calculated using MFPT and APTs using NDPT.

**Figure 3:**
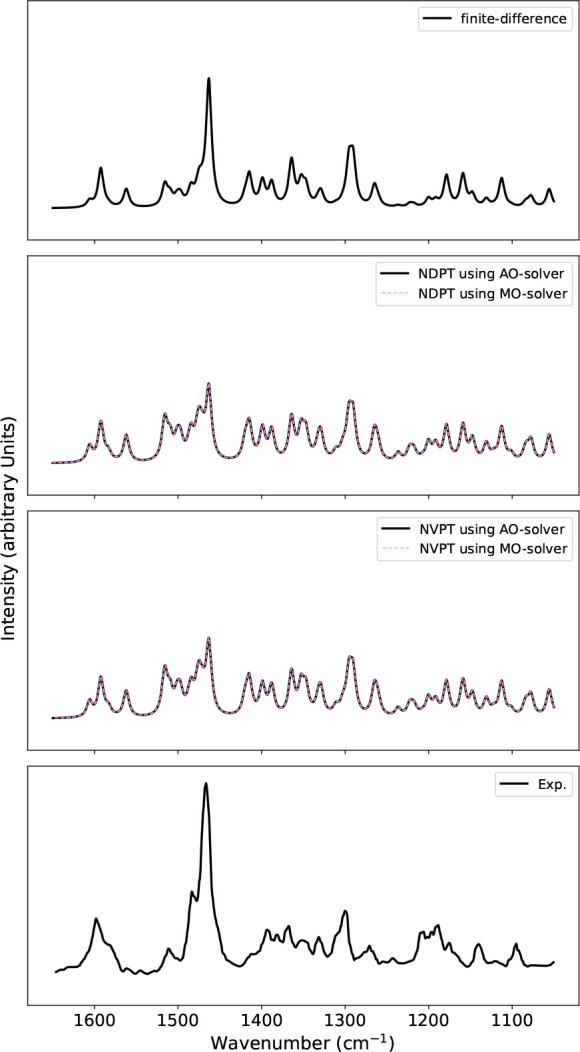
Plots illustrating the IR absorption spectra calculated using NDPT (second plot from the top) and NVPT (third plot) with MO-based and AO-based response solvers, respectively, compared with the spectrum calculated using the finite difference method (first plot) and the experimental spectrum (fourth plot) digitized from ref (52).

To validate the implementation of the AO-solver with MO-based solvers for VCD calculation, we considered an example of a single conformer of the R-enantiomer of mirtazapine whose IR and VCD spectra were previously studied in ref (52). We present the VCD spectra of the R-enantiomer using both NVPT and MFPT, and the IR spectra employing NDPT and NVPT. The spectra were calculated with both solvers. Available experimental spectra are also plotted in Figures 2 and 3. This work mainly focuses on the extension of the AO-based solver to VCD and IR spectra calculations and validation with respect to the previously implemented MO-based solver based on the Sternheimer equation approach. VCD calculations were checked for convergence with respect to charge density CUTOFF and DFPT convergence.

The mode frequencies calculated from vibrational analysis were shifted by 55 cm^–1^ toward higher wavenumbers to match the alignment of peak positions of the experimental spectra. The gas phase calculations of VCD spectra within the double harmonic approximation are plotted in Figure 2. It should be mentioned that the experimental spectra may contain noise, particularly in regions with low VCD intensity. Therefore, the focus is placed more on the overall pattern of band magnitudes. Experimental VCD and IR spectra of the (*R*)-enantiomer were measured in CDCl_3_ solution using an FT-VCD spectrometer, while theoretical spectra were calculated with the (*R*)-enantiomer in the gas phase.^52^

## 8 Results and Discussion

The comparison of experimental and calculated spectra for VCD using NVPT shows a rather close match. This includes matching both the intensity and the sign of the bands. Such agreement is crucial for determining the absolute configuration of a molecule. For detailed analysis, we refer to ref (52), because the present work focuses more on comparison of VCD calculations employing NVPT and MFPT with AO- and MO-based linear response solvers. The calculated MFPT spectra from MO-solver also show good agreement with NVPT spectra and experiment. Higher intensity peaks at around 1600, 1480, 1370, and 1275 cm^–1^ feature similar signs and band positions. However, the band near 1220 cm^–1^ shows a slight shift toward the lower wavenumber side, and small intensity peaks are observed with sign and intensity mismatches in the MFPT spectra when comparing with NVPT, which could be attributed to the numerical inaccuracies in the integration of Hartree and exchange-correlation potential.^15^ We carried out all VCD calculations using AO-solver as well and compared the results with the ones from the MO-based solver. Both solvers lead to comparable same spectra, confirming the validity and accuracy of the implementation.

As AO-based implementation was also extended to IR absorption spectra calculation, we calculated IR spectra for the same molecule using analogous input settings as used for VCD. As expected, IR spectra employing NDPT and NVPT using MO- and AO-based solvers exactly match and also show good agreement with experiment, as shown in Figure 3. We also show the numerically calculated IR calculation using finite differences in Figure 3. We observe some differences in the spectra obtained with finite difference and from the response solvers. Unlike the finite difference method, the analytical calculation is free from parameters such as step size and offers in principle higher accuracy. Further improvements to match the experimental conditions could be e.g. the inclusion of solvent and finite temperature effects such as done in molecular dynamics simulations at ambient conditions.^48,53,54^

CP2K can perform NDPT, NVPT, and MFPT calculations, with NDPT conducted first followed by the others, to simulate VCD spectra using two different approaches. Of these, NDPT calculations are the most time-consuming due to the real and symmetric perturbation Hamiltonian, requiring the solution of a coupled system of 3N_atoms_ response equations. NVPT, being an imaginary perturbation, involves solving uncoupled 3N_atoms_ response equations. On contrast to that, in MFPT, only three response equations corresponding to each Cartesian direction are solved. However, the APTs needed for VCD calculations are obtained from NDPT or NVPT requiring NDPT. Although both MFPT and NVPT involve an imaginary perturbation, resulting in uncoupled response equations, MFPT-based VCD spectra calculations are thus more efficient compared to NVPT-based calculations.

## 9 Conclusions

We presented the theory and implementation of the AO-based solver for VCD and IR spectra calculations for nonperiodic molecular systems in CP2K. Using the R-enantiomer of mirtazapine as an example, VCD spectra using both NVPT and MFPT methods and IR spectra using NDPT and NVPT were calculated with the results closely matching available theory and experimental spectra. Our analysis confirmed that the AO-solver produces spectra comparable to the MO-based solver. Based on previous studies,^37,38^ it is expected that the former will offer advantages in terms of computational efficiency (speed up, less memory requirement) for larger systems. In our recent work^38^ and another study,^37^ a detailed analysis of the scaling and memory demands of both response solvers have been presented. In ref (38), calculations for a system of up to 4096 water molecules, determining electric dipole–electric dipole polarizabilities using the velocity representation of the electric dipole operator, were performed. The AO-solver demonstrated better scalability, requiring less computational time as the system size increased compared to the MO-solver. Additionally, the AO-solver exhibited significantly lower random-access memory (RAM) demands, making it more efficient for large system calculations. Similar advantages of the use of the AO-based solver compared to the MO-based solver for VCD calculations can be expected and might be discussed in detail in future work.

The AO-solver demonstrated strong agreement with MO-solver in band intensities and positions for both VCD and IR spectra. To the best of our knowledge, this work presents the first implementation within an AO-based linear response framework for VCD spectra using MFPT and NVPT, as well as IR absorption calculations using NDPT and NVPT. The successful implementation and validation of this solver extend CP2K’s capabilities for calculating vibrational spectroscopy properties efficiently and accurately.

## Data Availability

Data supporting the results of this study are available from the corresponding author upon reasonable request.
